# A Nitrobenzoyl Sesquiterpenoid Insulicolide A Prevents Osteoclast Formation *via* Suppressing c-Fos-NFATc1 Signaling Pathway

**DOI:** 10.3389/fphar.2021.753240

**Published:** 2022-01-17

**Authors:** Yanhui Tan, Minhong Ke, Zhichao Li, Yan Chen, Jiehuang Zheng, Yiyuan Wang, Xuefeng Zhou, Gang Huang, Xiaojuan Li

**Affiliations:** ^1^ State Key Laboratory for Chemistry and Molecular Engineering of Medicinal Resources, Collaborative Innovation Center for Guangxi Ethnic Medicine, School of Chemistry and Pharmaceutical Sciences, Guangxi Normal University, Guilin, China; ^2^ Laboratory of Anti-inflammatory and Immunomodulatory Pharmacology, Guangdong Provincial Key Laboratory of New Drug Screening, School of Pharmaceutical Sciences, Southern Medical University, Guangzhou, China; ^3^ CAS Key Laboratory of Tropical Marine Bio-resources and Ecology, Guangdong Key Laboratory of Marine Materia Medica, South China Sea Institute of Oceanology, Chinese Academy of Sciences, Guangzhou, China; ^4^ Integrated Traditional Chinese and Western Medicine Hospital, Southern Medical University, Guangzhou, China

**Keywords:** insulicolide A, osteoclast, receptor activator of nuclear factor-κB ligand (RANKL), LPS, nuclear factor of activated T-cells cytoplasmic 1 (NFATc1)

## Abstract

It is a viable strategy to inhibit osteoclast differentiation for the treatment of osteolytic diseases such as osteoporosis, rheumatoid arthritis and tumor bone metastases. Here we assessed the effects of insulicolide A, a natural nitrobenzoyl sesquiterpenoid derived from marine fungus, on receptor activator of nuclear factor-κB ligand (RANKL)-stimulated osteoclastogenesis *in vitro* and its protective effects on LPS-induced osteolysis mice model *in vivo*. The results demonstrated that insulicolide A inhibited osteoclastogenesis from 1 μM *in vitro*. Insulicolide A could prevent c-Fos and nuclear factor of activated T-cell cytoplasmic 1 (NFATc1) nuclear translocation and attenuate the expression levels of osteoclast-related genes and DC-STAMP during RANKL-stimulated osteoclastogenesis but have no effects on NF-κB and MAPKs. Insulicolide A can also protect the mice from LPS-induced osteolysis. Our research provides the first evidence that insulicolide A may inhibit osteoclastogenesis both *in vitro* and *in vivo*, and indicates that it may have potential for the treatment of osteoclast-related diseases.

## Introduction

Bone resorption and formation are keeping a dynamic balance to maintain skeletal renewal and integrity. However, hyperactivity of osteoclast can lead to bone osteoclastic diseases such as osteoporosis, rheumatoid arthritis (RA), and tumor bone metastases (Perpetuo et al., 2017; Tsukasaki and Takayanagi, 2019; Győri and Mócsai, 2020; Kim et al., 2020; Liu et al., 2021). Therefore, targeting osteoclast formation has been regarded as a practicable treatment strategy to improve the prognosis of patients with bone destructive diseases (Broadhead et al., 2011; Stickeler and Fehm, 2014).

Osteoclasts, originate from bone marrow mononuclear macrophage lineage, are multinuclear and functioning as bone-resorbing cells (Boyle et al., 2003). Both receptor activator of nuclear factor-κB ligand (RANKL) and macrophage colony-stimulating factor (M-CSF) are essential for proliferation and differentiation of osteoclast (Arai et al., 1999; Cappellen et al., 2002; Lorenzo, 2017). Once RANKL is bound to its homologous receptor RANK, it first engages the adaptor protein tumor necrosis factor receptor-associated factor 6 (TRAF6) (Tanaka et al., 2005), then quickly triggers the signal cascade including nuclear factor NF-κB, mitogen-activated protein kinases (MAPKs) (Matsumoto et al., 2000; Li et al., 2003; Wada et al., 2006; Lee et al., 2016), followed by activating c-Fos (Grigoriadis et al., 1994). Activated NF-κB or c-Fos can induce the activation and amplification of the downstream nuclear factor of activated T-cell cytoplasmic 1 (NFATc1), which can initiate the expression of osteoclast-related genes including OSCAR, Blimp1, DC-STAMP, cathepsin K, TRAP, and so on (Takayanagi et al., 2002; Edwards and Mundy, 2011).

Natural products from marine fungus have become a rich source for developing novel drugs for the treatment of various diseases (Kang et al., 2015; Malve, 2016). Nitrobenzoyl sesquiterpenoids (NSs) represent a novel and rare class of compounds isolated from marine fungi, and only seven are identified until now (Wu et al., 2012; Zhao et al., 2016; Tan et al., 2018). We have identified one NS compound, 6*β*,9*α*-dihydroxy-14-*p*-nitrobenzoylcinnamolide (NS4), having the potential to suppress osteoclast formation by inhibiting NF-κB/RelB signaling pathway *via* binding to Arg B246 of NF-κB P65 (Tan et al., 2020). Another NS compound, insulicolide A, isolated from the marine-derived fungus *Aspergillus ochraceus*, has demostrated antiinflammation and antitumor activity *in vitro* (Wang et al., 2014; Guo et al., 2018). However, the influence of insulicolide A on osteoclast differentiation *in vitro* and bone lysis *in vivo* is not yet known.

Here, our study evaluated the suppressive effect of insulicolide A on RANKL-stimulated bone marrow monocytes (BMMs)-derived osteoclastogenesis *in vitro*, and tested the potential protective effects of insulicolide A on a LPS-induced osteolysis mice model *in vivo*. Insulicolide A mitigated osteoclastogenesis by preventing activation of c-Fos and NFATc1 but not affecting NF-κB signaling pathway.

## Methods

### Reagents and antibodies

Insulicolide A was extracted from the cultured *Aspergillus ochraceus* Jcma1F17 derived from marine fungus according to the methods described previously (Tan et al., 2018). The structure of the compound was identified by both high-resolution mass spectrometry (HRMS) and nuclear magnetic resonance (NMR). The purity of the sample was more than 95% as analyzed by high-performance liquid chromatography (HPLC). The compound was kept under −20°C for long-term storage and dissolved in dimethyl sulfoxide to a concentration of 10 mM as a reserve before usage. Our previous study described the extracting craft and structure of insulicolide A (Wu et al., 2012; Zhao et al., 2016; Tan et al., 2018). RAW264.7 cells stably transfected with luciferase reporter genes of NF-κB and NFATc1 is a gift from Professor Xu (University of Western Australia, Nedlands, Australia). Dulbecco’s modified Eagle’s medium (DMEM) and alpha minimum essential medium (*α*-MEM) were provided by Gibco (Rockville, MD, USA). Recombinant mouse M-CSF and RANKL are both from R&D (Minneapolis, Minnesota, USA). MTT, TRAP assay Kit, BAY11-7082, and CSA were all provided by Sigma- Aldrich (St. Louis, MO, USA). Osteo Assay Surface Polystyrene Microplates were provided by Corning (St. Lowell, MA, USA). qPCR Master Mix were obtained from Promega (United States). RNeasy kit and PrimeScript RT reagent kit were purchased from TaKaRa (China). Nuclear isolation kit was obtained from Cayman Chemicals (Ann Arbor, MI, USA). Rabbit mAbs of p65 (#49445), NFATc1 (#8032), p-ERK (#4370), ERK (#4695), p-p38 (#9215), p38 (#9255), p-JNK (#9255), JNK (#9215), c-Fos (#2250), *β*-actin (#3700), and murine mAb of lamin A/C (#4777) were all provided by CST (Beverly, MA, USA).

### Mice

Mice of C57BL/6J and ICR were provided by the medical animal center in Guangdong province, China. The mice were housed in an environment with the temperature of 22–24°C, a light/dark cycle of 12 h and 50–55% humidity. Water and food were provided at liberty. Animal studies were approved by the Committee of the animal protection and utilization of Southern Medical University and institutional animal protection and utilization of Guangxi Normal University.

### Cell culture

The marrow cavity of C57BL/6J mice with 6–8 weeks of age was exposed and flushed with sterile PBS under sterile conditions. Cells were then collected, and red blood cells were lysed accordingly. The obtained cells were incubated in *α*-MEM medium including 10% fetal bovine serum (FBS), 1% penicillin/streptomycin, and 50 ng/ml of M-CSF, and then nonadherent cells were collected for subsequent use. The mouse RAW264.7 cells transfected with luciferase reporter gene of NFATc1 were incubated in complete DMEM with 10% FBS in an incubator with 5% CO_2_ at 37°C.

### Cell viability assay

BMMs (1 × 10^3^ cells/well) with or without insulicolide A were treated in *α*-MEM medium supplemented with 50 ng/ml of M-CSF for 4 days. MTT assay was used to test cell viability following the instructions of the manufacturer.

### Osteoclastogenesis and tartrate-resistant acidic phosphatase assay

For osteoclastogenesis assay, BMMs (1 × 10^4^ cells/well) were first incubated with different levels of insulicolide A in a 96-well plate, then stimulated with 100 ng/ml of RANKL or 100 ng/ml of LPS and 50 ng/ml of M-CSF for 3 days. After that, the cells were fixed in 2.5% glutaraldehyde and then stained to assay tartrate-resistant acidic phosphatase (TRAP) activity. Images were taken, and quantitation of osteoclasts (nuclei > 5 for BMMs) were counted.

### Bone resorption pit assay

BMMs (1 × 10^4^ cells/well) were first incubated in Osteo Assay Surface Polystyrene Microplate, then administered with insulicolide A at different concentrations, stimulated by 50 ng/ml of M-CSF and 100 ng/ml of RANKL for 7 days. After that, 10% bleach solution was used to wash the cells. The resorption areas of osteoclast were quantified by Image-Pro Plus 6.0.

### Nuclear factor of activated T-cell cytoplasmic 1 luciferase reporter assay

The activity of luciferase reporter gene of NFATc1 induced by RANKL was measured as mentioned earlier. RAW264.7 cells, which were transfected with a NFATc1-responsive luciferase construct, were pretreated with insulicolide A and CsA (NFATc1 inhibitor, 1 μM) for 4 h. After incubated by 100 ng/ml of RANKL for 12 h, the activity of luciferase was assayed.

### Quantitative real-time polymerase chain reaction

Briefly, BMMs (1 × 10^6^ cells/ml) were treated by insulicolide A at different levels for 4 h, followed by stimulation by RANKL and M-CSF for 24 h. The RNeasy mini kit was first used to isolate the total RNA, then, PrimeScript RT kit was prepared for synthesis of cDNA. Real-time PCR was performed using qPCR Master Mix. Polymerase chain reaction was executed by a procedure of 95°C (30 s), 95°C (5 s), and 60°C (34 s) in 40 cycles. The mouse primers used are as follows: DC-STAMP (forward: AGA​CGT​GGT​TTA​GGA​ATG​CAG​CTC; reverse: TCC​TCC​ATG​AAC​AAA​CAG​TTC​CAA), cathepsin K (forward: GGC​CAA​CTC​AAG​AAG​AAA​AC; reverse: GTG​CTT​GCT​TCC​CTT​CTG​G), GAPDH (forward: ACA​CAT​TGG​GGG​TAG​GAA​CA; reverse: AAC​TTT​GGC​ATT​GTG​GAA​GG), OSCAR (forward: CCT​AGC​CTC​ATA​CCC​CCA​G; reverse: CGT​TGA​TCC​CAG​GAG​TCA​CAA). The comparative 2^−ΔΔ*CT*^ method was used to calculate the relative expression of target genes. The mean Ct value of target genes in the experimental groups were normalized to the Ct values of GAPDH.

### Western blot analysis

BMMs (1 × 10^6^ cells/ml) were treated with different concentrations of Insulicolide A for 4 h, then incubated with 100 ng/ml RANKL for additional 30 min or 24 h. Total proteins were extracted by using RIPA buffer and cytoplasmic and nuclear proteins were prepared with a nuclear extraction kit. Proteins were separated on SDS-PAGE, and then transferred to PVDF membranes. The primary antibodies were used to incubate with membranes at 4°C overnight after blocked by 5% non-fat milk for 1 h. On the next day, TBST was used to wash the membranes and then the secondary antibodies were used to incubate for another 1 h at room temperature. Finally, the blotted protein bands were obtained with a chemilunimescence kit (Yeasen Biotech, China) and quantification of the band intensities was analyzed by ImageJ software. The expression of p65, p38, ERK, JNK, p-ERK, p-p38, p-JNK were measured after treatment of RANKL for 30 min and the expression of c-Fos, NFATc1, DC-STAMP were measured after RANKL treatment for 24 h.

### LPS-induced murine inflammatory osteolysis model *in vivo*

Female ICR mice aged 8–9 weeks were randomly divided into four groups with six mice in each group: the control group (served with PBS), model group (served with LPS), model with low dose Insulicolide A (5 mg/kg), and model with high dose Insulicolide A (10 mg/kg). LPS administration was by intraperitoneal injection (5 mg/kg body weight) on days 1 and 4. Insulicolide A or PBS was administered once daily *via* gavage for 8 days. Eight days later, the ICR mice were euthanized. The left femur of all the animals were obtained and scanned by a micro-CT (CT80, ScancoMedical, Zurich, Switzerland) with the following instrument parameters: 50 kV, 500 μA, and 0.7° rotation step. The parameters of trabecular bone contains the ratio of bone volume to tissue volume (BV/TV), trabecular number (Tb.N), Mean density of TV and trabecular separation (Tb.Sp). Removal of the right femur from experimental mice to fix in 4% PFA at 4°C for 24 h, and then the femurs embedded in paraffin after decalcification in 12% EDTA for 1 month were sectioned for H&E and TRAP. As for the H&E and TRAP staining images, there were *n* = 12 images taken in total per group (two images from each mouse). A 600-μm × 600-μm region of interest located 150 μm below the growth plate of the femur metaphysis was employed for the assessment of the number of TRAP-positive multinucleated cells and osteoclastic surface/bone surface (Oc.S/BS). Histomorphometry analysis was performed using Image Pro Plus 6.0 (IPP) software.

### Data analysis

All data were expressed as the mean ± standard deviation of three or more experiments. For multiple comparisons, differences were tested using a regular one-way ANOVA, followed by a Tukey multiple comparison for groups with a Gaussian distribution or with a Friedman test, followed by a Dunn’s posttest for multiplicity if a Gaussian distribution could not be assumed. The *p*-values of less than 0.05 were deemed to be statistically significant.

## Results

### Insulicolide A inhibited receptor activator of nuclear factor-κB ligand-induced osteoclastogenesis in bone marrow monocytes *in vitro*

**FIGURE 1:**
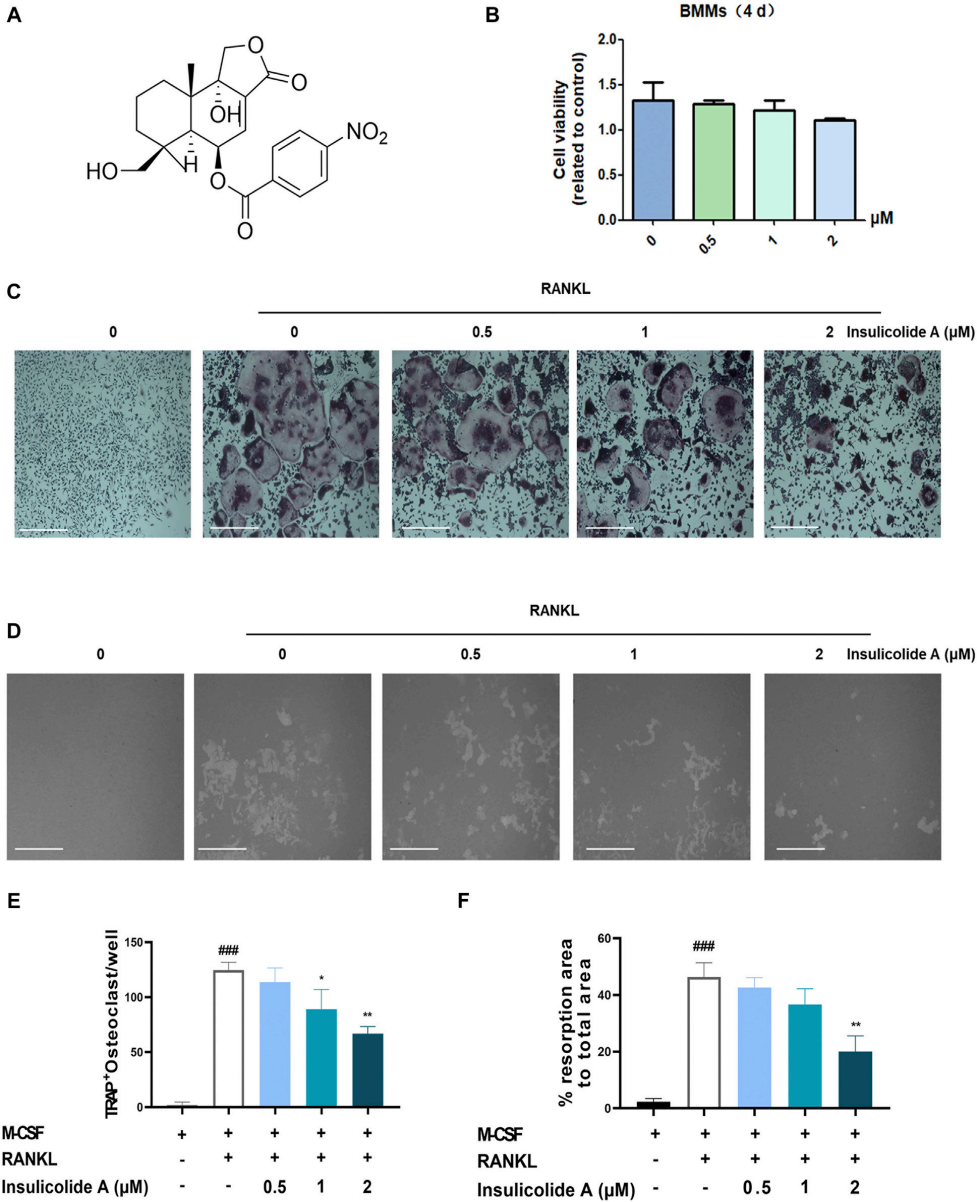
Insulicolide A inhibited receptor activator of nuclear factor-κB ligand (RANKL)-induced osteoclastogenesis *in vitro*. **(A)** The chemical structure of insulicolide A. **(B)** The cell viability of 0.5–2 μM insulicolide A in bone marrow monocytes (BMMs) for 4 days were measured. **(C, E)** Images and number of TRAP-positive multinucleated cells (nuclei > 5) were taken and calculated. **(D, F)** Images and areas of bone resorption by osteoclasts on the hydroxyapatite-coated surfaces were taken and quantified. The data are shown as means ± SD (*n* = 3 independent experiments, containing three replicate samples each). ^###^ *p* < 0.001 vs. nontreatment groups, *p < 0.05, **p < 0.01 vs. RANKL-induced groups.

To detect the effects of insulicolide A (Figure 1A) on RANKL-induced osteoclastogenesis, BMMs were first incubated with different concentrations of insulicolide A from 0.5 to 2 μM, then followed by incubation of RANKL and M-CSF. BMMs can differentiate into TRAP-positive osteoclasts in the presence of RANKL (Figures 1C, E). However, insulicolide A remarkably reduced osteoclastogenesis induced by RANKL in a dose-dependent manner without cytotoxicity (Figures 1B– C, E). Additionally, we found that insulicolide A also decreased LPS-induced osteoclastogenesis (Supplementary Figures S1A, B). Bone erosion is caused by the increased number of bone-resorbing osteoclasts, so we next evaluated the influence of insulicolide A on bone erosion of BMMs on hydroxyapatite-coated plates. Similar results were obtained, that reduced erosion areas was consistent with the reduced osteoclast number by insulicolide A, and the erosion areas were almost completely decreased by insulicolide A at 2 μM (Figures 1D, F).

Insulicolide A had no statistically significant effects on activation of RANKL-induced NF-κB and MAPKs.

**FIGURE 2:**
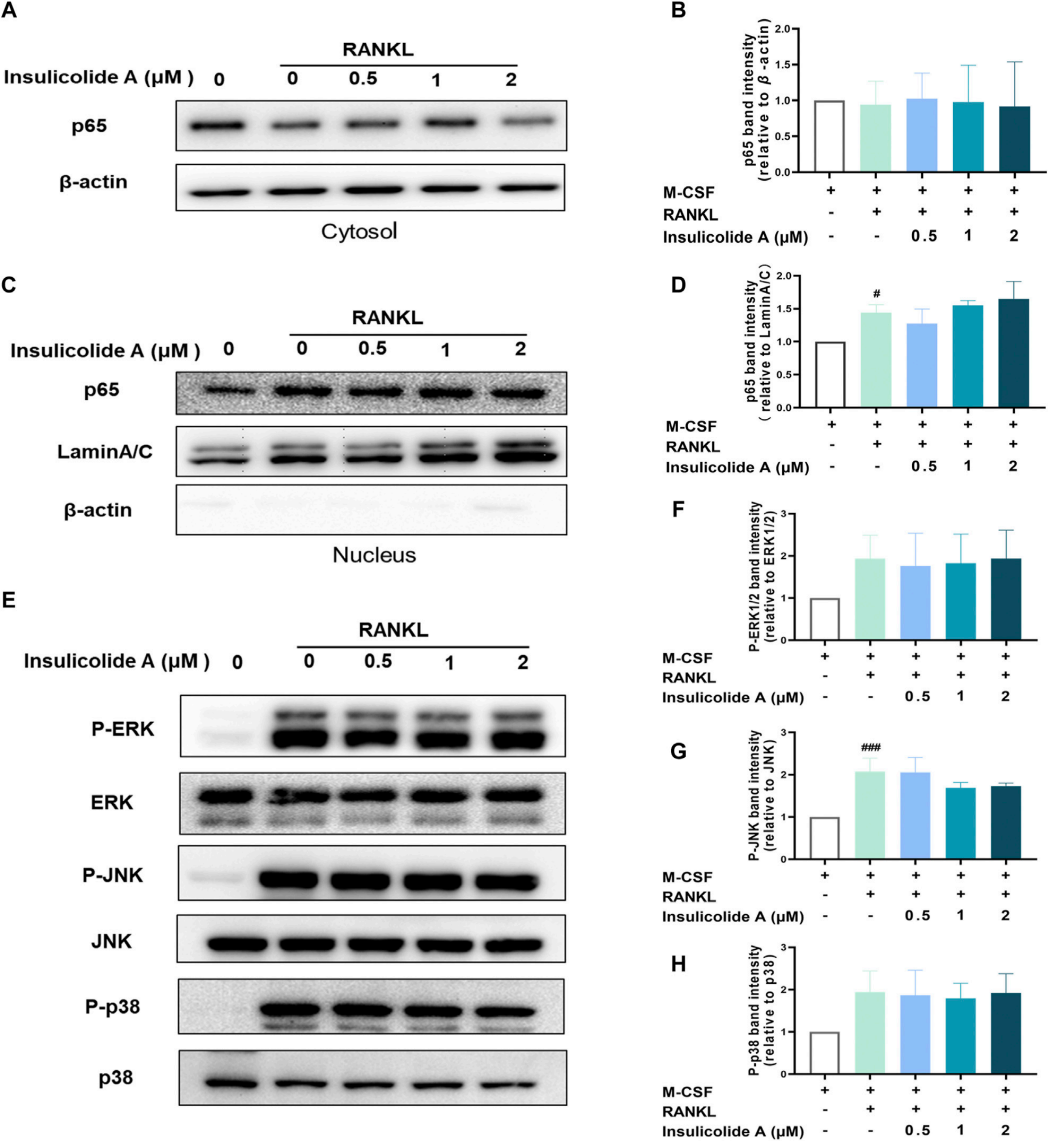
Insulicolide A had little influence on activation of RANKL-induced NF-κB and mitogen-activated protein kinases (MAPKs). Insulicolide A (0.5–2 μM) in BMMs were first cultured for 4 h, then followed by 100 ng/ml of RANKL stimulation for 30 min. After that, the proteins were harvested and detected by p65, LaminA/C, *β*-actin, ERK, P-ERK, JNK, P-JNK, p38, and P-p38 antibodies. The expression of nuclear or total protein levels of p65 **(A, B)** to *β*-actin, or p65 **(C, D)** to LaminA/C, and P-ERK to ERK, P-JNK to JNK or P-p38 to p38 **(E–H)** were determined by ImageJ software. The data are shown as means ± SD of three independent experiments. ^#^ *p* < 0.05, ^###^ *p* < 0.001 vs. nontreatment groups.

Since we found insulicolide A attenuated osteoclast formation, we next elucidated the mechanisms of insulicolide A during RANKL-induced osteoclastogenesis. As NF-κB and MAPKs signaling pathways are important in RANKL-induced osteoclast formation, we first investigated the effects of insulicolide A on RANKL-induced NF-κB activation including the protein expressions of NF-κB p65 in cytosol and nucleus. Here, Western blotting assays indicated that the nuclear protein expression of NF-κB p65 increased remarkably after RANKL stimulation; however, insulicolide A from 0.5 to 2 μM showed no statistically significant effects on p65 nuclear translocation (Figures 2A–D).

Then, we determined whether insulicolide A could attenuate the activation of MAPKs containing ERK/P-ERK, p38/P-p38, and JNK/P-JNK during RANKL-induced osteoclastogenesis. The protein expressions of P-ERK, P-p38, and P-JNK were enhanced rapidly after stimulation with RANKL; however, insulicolide A had no statistically significant effect on the phosphorylation of ERK, p38, and JNK, which was consistent with the influence on NF-κB (Figures 2E–H). The above results suggested that insulicolide A inhibits RANKL-induced osteoclast formation through other signaling pathways rather than NF-κB or MAPKs.

### Insulicolide A suppressed receptor activator of nuclear factor-κB ligand-induced c-Fos/nuclear factor of activated T-cell cytoplasmic 1 signaling pathway

**FIGURE 3:**
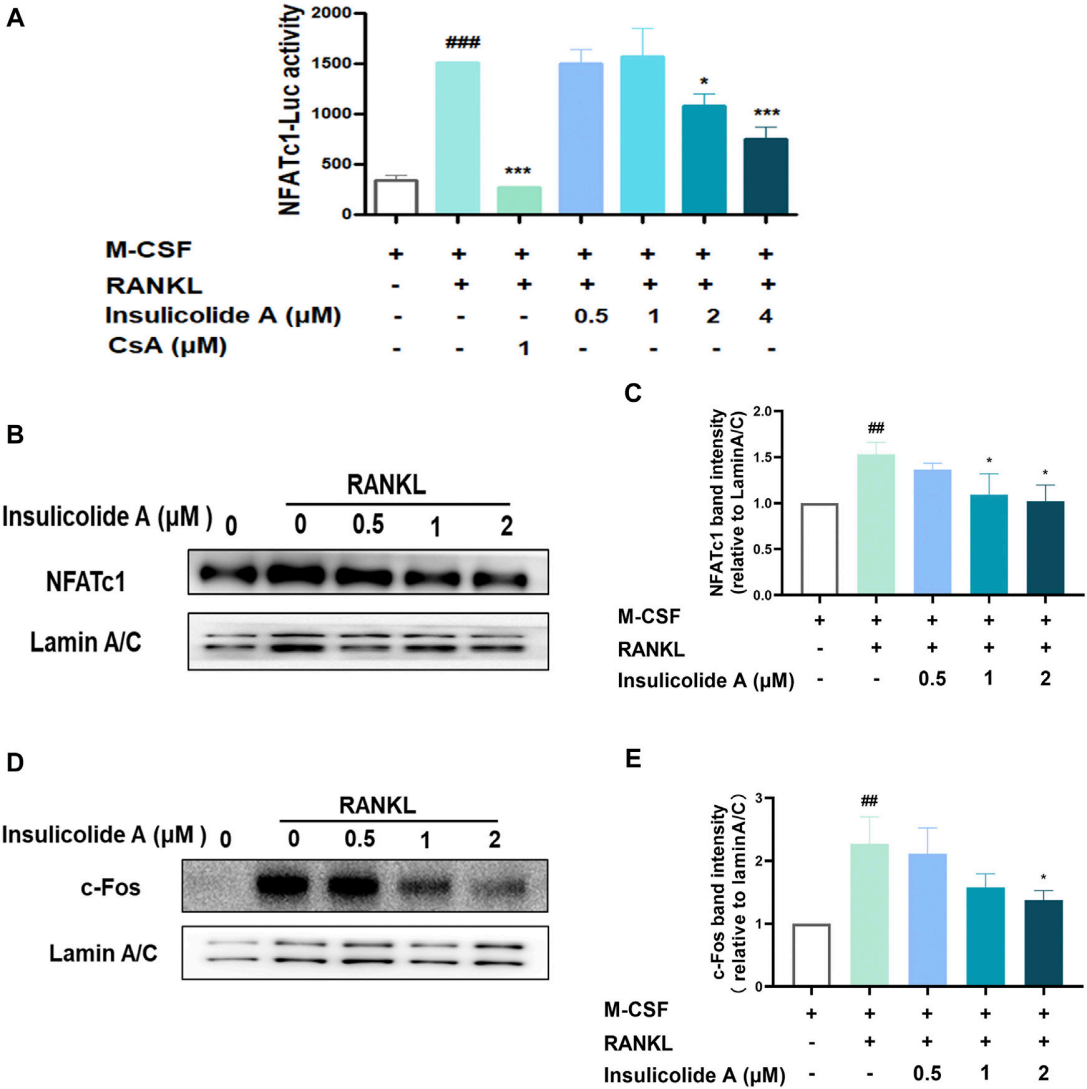
Insulicolide A inhibited RANKL-induced c-Fos/nuclear factor of activated T-cells cytoplasmic 1 (NFATc1) signaling pathway. RAW264.7 cells, stably transfected by NFATc1 luciferase reporter vector, were first pretreated by insulicolide A (0.5–4 μM) for 4 h, then followed by 100 ng/ml of RANKL stimulation for 6 h, and the luciferase activity **(A)** was assayed later. Insulicolide A (0.5–2 μM) in BMMs were first cultured for 4 h, then followed by 100 ng/ml of RANKL stimulation for 30 min. After that, nuclear proteins were harvested and detected by NFATc1, c-Fos, and LaminA/C antibodies. The relative expression of **(B, C)** NFATc1 to LaminA/C or **(D, E)** c-Fos to LaminA/C were analyzed. The data are shown as means ± SD of three independent experiments. ^##^ *p* < 0.01, ^###^ *p* < 0.001 vs. nontreatment groups, *p < 0.05, ***p < 0.001 vs. RANKL-induced groups.

NFATc1, the master nuclear transcription factor, can activate osteoclastogenesis, and the activation of NFATc1 depends on NF-κB, MAPKs signaling pathways, or its upstream transcriptional regulator c-Fos. Since insulicolide A has little effect on both RANKL-induced NF-κB and MAPKs, we next examined the influence of insulicolide A on the activation of nuclear transcription factor c-Fos and NFATc1. First, we used NFATc1 luciferase reporter assay to determine the influence of insulicolide A on NFATc1 activation, and found that RANKL-induced NFATc1 luciferase activity was markedly suppressed by insulicolide A at 2 and 4 μM concentrations (Figure 3A). Then Western blot assay revealed that the nucleus protein expression of RANKL-induced NFATc1 was increased. However, insulicolide A abrogated nucleus protein expression of NFATc1 in a dose-dependent manner (Figures 3B, C). NFATc1 self-amplification and activation are produced by the binding of up-stream nuclear transcription factor c-Fos, which combines with the NFATc1 promoter. C-Fos knockout mice showed severe osteosclerosis due to the reduced osteoclasts. In line with NFATc1, c-Fos protein levels were also restrained by insulicolide A (Figures 3D, E).

Together, our results demonstrated that insulicolide A might attenuate RANKL-induced osteoclast formation by targeting the c-Fos/NFATc1 signaling pathway.

Insulicolide A attenuated the expression of osteoclast relative genes and DC-STAMP induced by RANKL.

**FIGURE 4:**
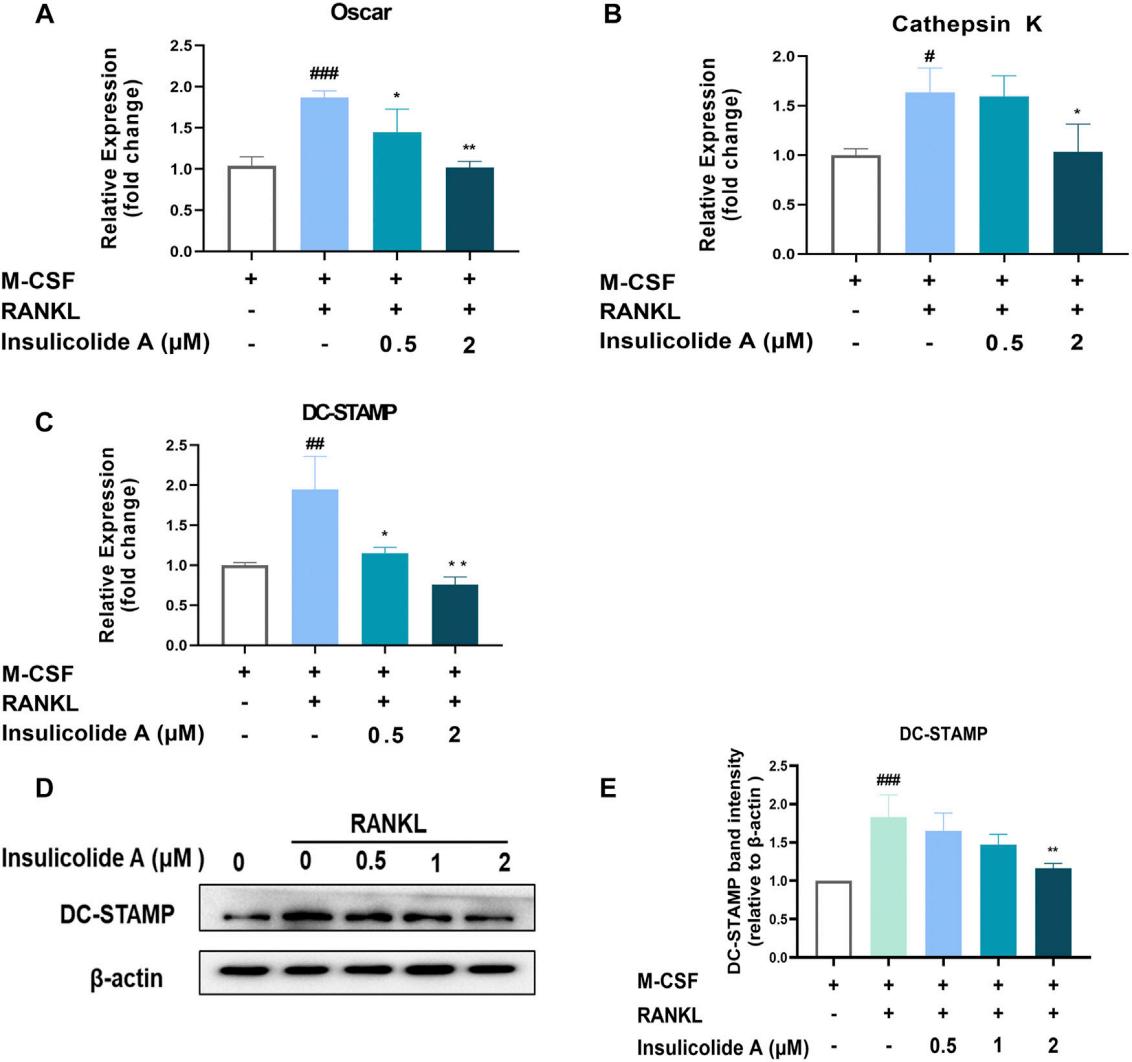
Insulicolide A attenuated the expression of osteoclast-related genes and DC-STAMP induced by RANKL. Insulicolide A (0.5–2 μM) in BMMs were first cultured for 4 h, then followed by 100 ng/ml of RANKL stimulation for 24 h. After that, real-time PCR was used to analyz the gene expression of OSCAR **(A)**, cathepsin K **(B)**, and DC-STAMP **(C)**. Antibodies were used to detect the total proteins expression of DC-STAMP and *β*-actin **(D)**. **(E)** The relative expression of DC-STAMP to *β*-actin was analyzed by ImageJ software. The data are shown as means ± SD of three independent experiments. ^#^ *p* < 0.05, ^##^ *p* < 0.01, ^###^ *p* < 0.001 vs. nontreatment groups, *p < 0.05, **p < 0.01 vs. RANKL-induced groups.

Once NFATc1 is activated, osteoclast formation related genes, such as TRAP, OSCAR, cathepsin K, and osteoclast function-related genes including DC-STAMP, were strongly enhanced. However, when treated with insulicolide A, the mRNA expression of OSCAR, cathepsin K, and DC-STAMP were inhibited remarkably (Figures 4A–C). DC-STAMP, a multi-pass transmembrane molecule, is essential for the fusion and resorptive capacity of preosteoclasts. In DC-STAMP-deficient mice, the number of multinucleated osteoclasts reduced and bone mineral density increased. We then further examined the protein levels of DC-STAMP, consistent with the mRNA levels, insulicolide A also inhibited the protein levels of DC-STAMP induced by RANKL (Figures 4D, E). Collectively, these results suggested that insulicolide A inhibited RANKL-induced osteoclastogenesis and bone resorptive function by decreasing osteoclast formation-related genes and fusion-related genes.

### Insulicolide A decreased LPS-induced bone loss by inhibiting osteoclast activity

**FIGURE 5:**
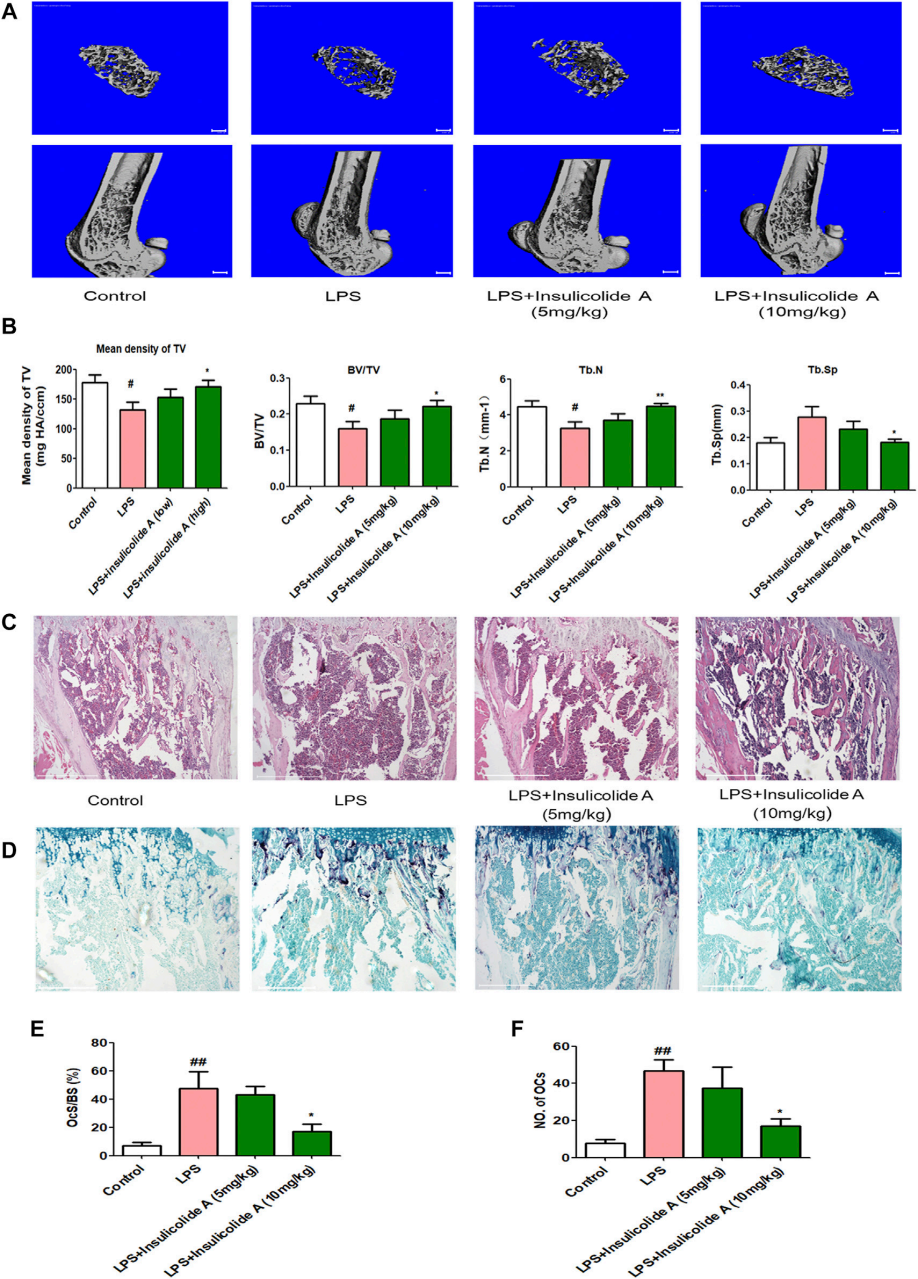
Insulicolide A decreased LPS-induced murine bone loss by suppressing osteoclast activity. All mice were randomly divided into four groups with six mice in each group: control group (injected with PBS), LPS group (injected with LPS), LPS + insulicolide A (5 mg/kg) group, and LPS + insulicolide A (10 mg/kg) group. **(A)** Representative 3D reconstruction micro-CT images of transverse (above) and longitudinal (below) sections of the femur in different groups. **(B)** Quantitative measurements of trabecular bone parameters containing BMD, BV/TV, Tb.N, and Tb.Sp. Representative H&E **(C)** and TRAP staining **(D)** images of the femurs in each group. Osteoclast surface/bone surface **(E)** and the number of osteoclasts in each group **(F)** were analyzed. *n* = 6 mice per group. ^#^ *p* < 0.05, ^##^ *p* < 0.01 vs. PBS-treatment groups, *p < 0.05, **p < 0.01 vs. LPS-treatment groups.

LPS, an effective endotoxin from the cell wall in Gram-negative bacteria, can directly induce osteoblasts to secrete RANKL and then activate osteoclast formation. The activation of osteoclasts can result in osteoclastic diseases. In order to investigate the role of insulicolide A in the suppression of osteoclast function *in vivo*, LPS-induced inflammation bone loss mice model was used. Micro-CT assays showed that LPS-injected mice model suffered a serious bone loss; however, when LPS-injected mice were treated with insulicolide A by gavage, both low-dose group (5 mg/kg) and high-dose group (10 mg/kg) could attenuate LPS-induced bone destruction (Figure 5A). Bone parameter analysis indicated that compared with LPS-injected mice, mean density of TV, BV/TV, Tb.N of insulicolide A (10 mg/kg)-treated mice were markedly increased, but Tb.Sp was decreased (Figure 5B). H&E-stained bone sections further confirmed the protection of insulicolide A on LPS-induced bone loss. Furthermore, TRAP staining assays and histomorphometric analysis of the number of osteoclasts and the percentage osteoclast surface per bone surface (OcS/BS) in the bone trabecula demonstrated that oral treatment of insulicolide A could remarkably decrease LPS-caused bone destruction and inhibited osteoclast numbers (Figures 5C–F). Taken together, our results indicated that oral administration of insulicolide A could prevent inflammatory bone destruction *in vivo*.

## Discussion

Sesquiterpenoids from the plants and microorganisms display various biological activities. Marine fungus-derived nitrobenzoyl sesquiterpenoids, rare from natural source, exhibit remarkable pharmacological activities, including antitumor and antiinflammation (Wang et al., 2014; Guo et al., 2018). Here our results indicated that marine-derived nitrobenzoyl sesquiterpenoid, insulicolide A, could attenuate osteoclastogenesis c-Fos-NFATc1 signaling pathway induced by RANKL *in vitro* at doses from 1 to 2 μM. Consistently, we also found that insulicolide A could protect inflammatory osteolysis *in vivo*.

During the process of RANKL-induced osteoclast formation, the combination of RANKL to RANK can recruit TRAF6 followed by activation of NF-κB and MAPKs signaling pathways. NF-κB and MAPKs are crucial pathways of RANKL response in osteoclastogenesis (Ghosh and Karin, 2002; Jimi and Ghosh, 2005). However, our data showed that insulicolide A had no significant reduction on p65 of NF-κB and the phosphorylation of ERK, p38, and JNK of MAPKs, which were incompletely consistent with the effects of NS4 (structural isomer of insulicolide A) on NF-κB. NS4 exhibited inhibitory effects on NF-κB by binding with NF-κB p65 Arg B246 (Tan et al., 2020), the different mechanisms between NS4 and insulicolide A on NF-κB may be due to their different conformations and their affinity with NF-κB. These results suggested that NF-κB and MAPKs might not be a downstream signal by insulicolide A to treat RANKL-induced osteoclast differentiation.

C-Fos, the subunit of activator protein-1 (AP-1), is induced during the process of RANKL-induced osteoclastogenesis (Grigoriadis et al., 1994). As the upstream nuclear transcription factor of NFATc1, activated c-Fos can bind to the promoter region of NFATc1 resulting in the autoamplification and activation of NFATc1 (Takatsuna et al., 2005). C-Fos knockout mice showed decreased NFATc1 nuclear translocation and severe bone sclerosis (Matsuo et al., 2004). Here, our findings determined that the nuclear protein level of c-Fos was drastically inhibited by insulicolide A in the BMMs of RANKL stimulation, which was also different with NS4, while NS4 had little effect on c-Fos nuclear expression during RANKL-induced osteoclast formation (Tan et al., 2020). Thus, the decrease in RANKL-induced c-Fos activation and the following attenuated NFATc1 nuclear protein levels, contributes to impaired osteoclastogenesis after insulicolide A treatment.

NFATc1 is the core transcriptional switch of osteoclast terminal differentiation (Nepal et al., 2013). Activated NFATc1 not only increases the expression levels of osteoclast-related genes including TRAP, OSCAR, and cathepsin K, but also refers to the multinucleation of osteoclast precursors through cell fusion proteins such as DC-STAMP (Yagi et al., 2006; Chiu and Ritchlin, 2016). In our study, the levels of OSCAR and cathepsin K induced by RANKL were remarkably reduced after treatment of insulicolide A. Additionally, RANKL-induced mRNA and protein expressions of DC-STAMP in osteoclast formation were also down-regulated after treatment with insulicolide A. The reduction of nuclear translocation of NFATc1 and the suppression of DC-STAMP caused by insulicolide A might attenuate the expression of osteoclast-specific genes and the fusion of preosteoclast cells to inhibit osteoclasts *in vitro*.

LPS is an effective endotoxin from the cell wall in Gram-negative bacteria, which has an important impact on bone loss (Place et al., 2021). In our experiment, we constructed murine bone loss model induced by LPS to evaluate the influence of insulicolide A on osteoclastogenesis *in vivo*. Micro-CT analysis indicated that oral insulicolide A (10 mg/kg) could recover bone loss induced by LPS through promoting BMD, BV/TV, and Tb.N, while reducing Tb.Sp. H&E and TRAP-stained bone section analysis further showed that insulicolide A remarkably inhibited bone destruction and osteoclast numbers in LPS-induced mouse model. Therefore, these results further manifested that oral insulicolide A protected bone by decreasing the numbers of osteoclast and improving bone parameters *in vivo*, which were consistent with its inhibitory effects on osteoclasts *in vitro*.

The up-stream signals such as TRAF6, DC-STAMP, and NF-κB/MAPKs regulate the expression of c-Fos during osteoclastogenesis (Yin et al., 2017; Liu et al., 2021; Zou et al., 2021). Here, insulicolide A showed little effect on NF-κB/MAPKs. DC-STAMP knockdown decreases c-Fos and NFATc1 expression in osteoclast precursor cells (Yin et al., 2017), thus, DC-STAMP can also serve as the surface molecule for insulicolide A to suppress osteoclast formation. Furthermore, the analysis of proteins binding with insulicolide A in osteoclastogenesis can be performed to disclose the target of insulicolide A in the future. In addition, we found that insulicolide A protected bone only by resorption *in vivo*. Further studies aimed at bone formation in osteoclast-related mouse models may make a better understanding of this compound for its treatment of osteolytic diseases.

In conclusion, this study discovered that insulicolide A, a natural nitrobenzoyl sesquiterpenoid isolated from the marine-derived *Aspergillus ochraceus* fungus, can suppress RANKL-induced osteoclastogenesis by preventing c-Fos rather than NF-κB and MAPKs, and then decreasing the level of NFATc1 and DC-STAMP *in vitro*. Our data, including the murine femur model experiment, suggest insulicolide A as a substantial osteoclasts inhibitor and indicate its promising application for osteoclast overactivated diseases.

## Data Availability

The original contributions presented in the study are included in the article/Supplementary Material, further inquiries can be directed to the corresponding authors.
