# Supplementary material for: A Nitrobenzoyl Sesquiterpenoid Insulicolide A Prevents Osteoclast Formation via Suppressing c-Fos-NFATc1 Signaling Pathway
Source: Front Pharmacol. 2022 Jan 17;12:753240. doi: 10.3389/fphar.2021.753240 (PMC8801808; doi:10.3389/fphar.2021.753240)
Supplement: Supplementary file 1 [file DataSheet1.docx]

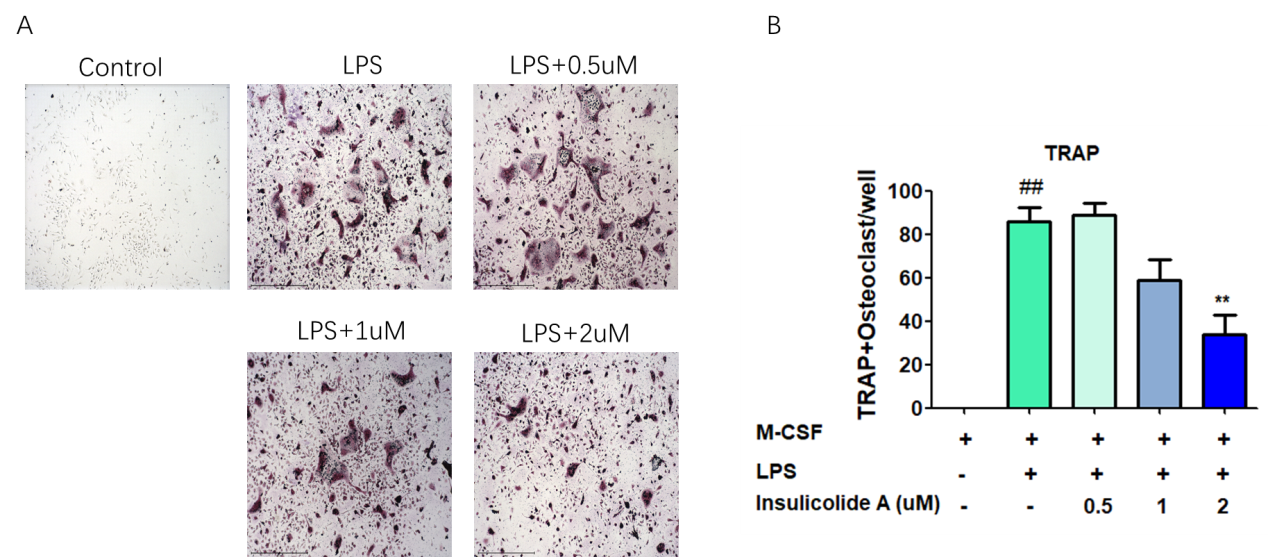


Figure S1. Insulicolide A inhibited LPS-induced osteoclastogenesis in vitro. (A) (B) Images and number of TRAP-positive multinucleated cells (nuclei > 5) were taken and calculated. The data are shown as means ± SD (of 3 independent experiments). ^##^p < 0.01 vs non-treatment groups, **p < 0.01 vs LPS-induced groups.


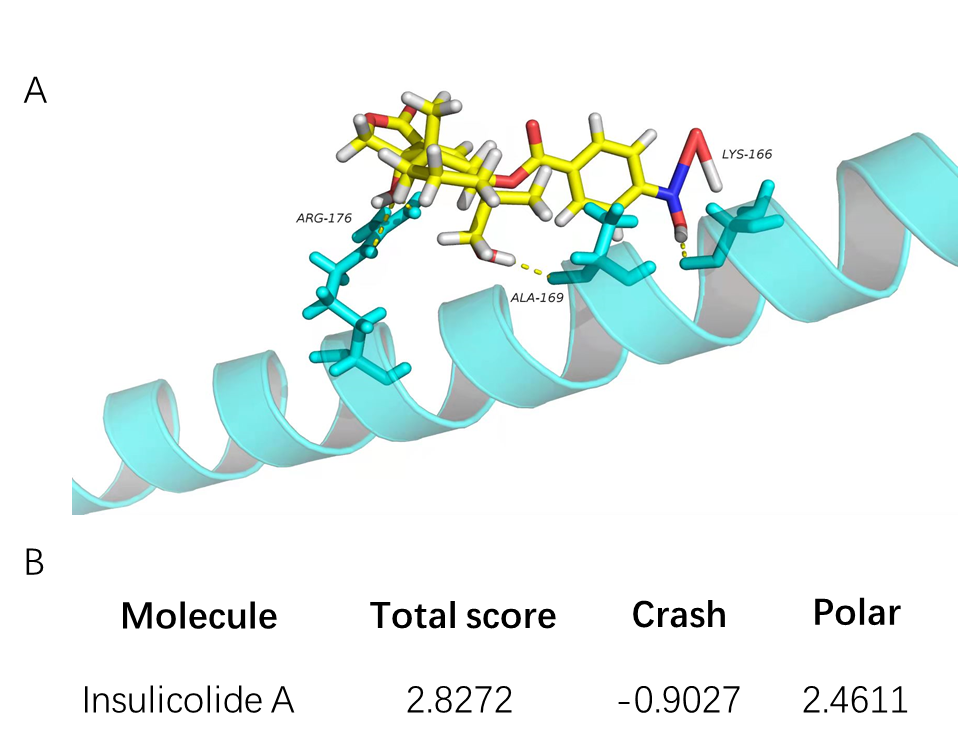


Figure S2. Molecular docking of Insulicolide A with c-Fos. (A) Binding sites of the molecule Insulicolide A with c-Fos protein. (B) The interaction details of the predicted binding mode of Insulicolide A with c-Fos.
